# Brachydactyly Mental Retardation Syndrome Diagnosed in Adulthood

**DOI:** 10.7759/cureus.3169

**Published:** 2018-08-21

**Authors:** Rupak Mahendhar, Paria Zarghamravanbakhsh, Maia Natalia Pavlovic, Radu Butuc, Issac Sachmechi

**Affiliations:** 1 Internal Medicine, Icahn School of Medicine at Mount Sinai/Queens Hospital Center, New York, USA; 2 Endocrinology, Icahn School of Medicine at Mount Sinai Queens Hospital Center, New York, USA; 3 Diabetes and Endocrinology, Icahn School of Medicine at Mount Sinai/Queens Hospital Center, Jamaica , USA; 4 Hospitalist Adult Internal Medicine, Presbyterian Hospital, Albuquerque, USA

**Keywords:** brachydactyly, 2q37 deletion syndrome, albright hereditary osteodystrophy

## Abstract

Brachydactyly mental retardation syndrome (BDMR) is due to a rare, small chromosomal deletion of 2q37, and manifests with variable signs and symptoms in people who live with it. BDMR could be misdiagnosed as Albright hereditary osteodystrophy (AHO), because it presents with lack of hormone resistance to parathyroid hormone (PTH) and similar skeletal and craniofacial abnormalities; however, BDMR is far rarer and can present with a different phenotype. In some cases, BDMR patients exhibit malformations of the internal organs, which could cause life-threatening health issues. Associations have also been made between this chromosomal deletion and autism as well. We here report a case of BDMR with an AHO-like phenotype: mild mental retardation, along with normal calcium, phosphate, and PTH levels. Since our patient had a normal biochemical test, we considered pseudopseudohypoparathyroidism (PPHP) as the diagnosis and genetic testing was performed. Karyotype analysis showed deletion of the long q-arm of chromosome 2 in all analyzed cells-46 XX, del (2)(q37.1), which was consistent with BDMR. This deletion is a loss of around 100 genes that can present itself in various ways neurologically and physiologically, depending on the genes lost. However, because patients experience a range of symptoms such as autism, seizures, heart defects, brachydactyly, there could be unforeseen complications with BDMR. Therefore, we postulate that it is necessary to consider a diagnosis of BDMR in adults with AHO-like phenotype and normal calcium metabolism.

## Introduction

Brachydactyly mental retardation syndrome (BDMR) is a rare disease with roughly 100 cases reported worldwide. Patients with BDMR present with a phenotype similar to Albright hereditary osteodystrophy (AHO) but without hormone resistance, hence a normal calcium metabolism. AHO occurs due to an inactivating mutation in the GNAS gene which is a G protein complex involved in signal transduction. Pseudohypoparathyroidism (PHP) and pseudopseudohypoparathyroidism (PPHP) are variants of AHO. Pseudo-hypoparathyroidism is characterized by AHO phenotype, low calcium, and high potassium levels. The biochemical abnormality is due to the resistance of end-organs to parathyroid hormone. This resistance will be evident when the defect is maternally inherited. Patients with PPHP have similar AHO phenotype but without the biochemical abnormality and the defect is paternally inherited [1]. We here present a case of a 45-year-old female with AHO-like phenotype, mild mental retardation but normal calcium and phosphate level whereas the abstract of this case has already been presented as an oral presentation (Abstract: Butuc R, Galibov R, Sachmechi I. Brachydactyly Mental Retardation Syndrome Diagnosed in Adulthood; 2014; https://www.aace.com/files/late-breaking-abstracts-2014.pdf).

## Case presentation

A 45-year-old female presented to Endocrinology clinic for evaluation of weight gain. During the interview, she reported polyphagia and polydipsia. Her past medical history was significant for hypertension, hyperlipidemia, obesity, sleep apnea, peripheral vascular disease and mood disorder. She was also diagnosed with mild mental retardation as a child. She experienced her menarche at the age of 13 and had regular periods. She was living in a group home and was not married or had kids. The patient’s height was 4 feet 11 inches and she had a body mass index (BMI) of 40. Physical examination was significant for short stature, facial dysmorphism with prominent forehead, upslanted eyes, flat nasal bridge and a thin upper lip. Extremity examination revealed short 4th and 5th metacarpal and metatarsal bones bilaterally.

Secondary causes of obesity and AHO were considered in differential diagnosis. On blood testing the patient’s calcium, phosphate and parathyroid hormone (PTH) levels were normal. Cortisol, thyroid stimulating hormone (TSH), and free thyroxine (FT4), follicle stimulating hormone (FSH) and luteinizing hormone (LH) levels were all normal. The patient was sent for genetic testing with a presumed diagnosis of PPHP. Karyotype test showed terminal deletion of the long q-arm of one chromosome 2 in all analyzed cells-46, XX, del (2)(q37.1), consistent with BDMR.

**Figure 1:**
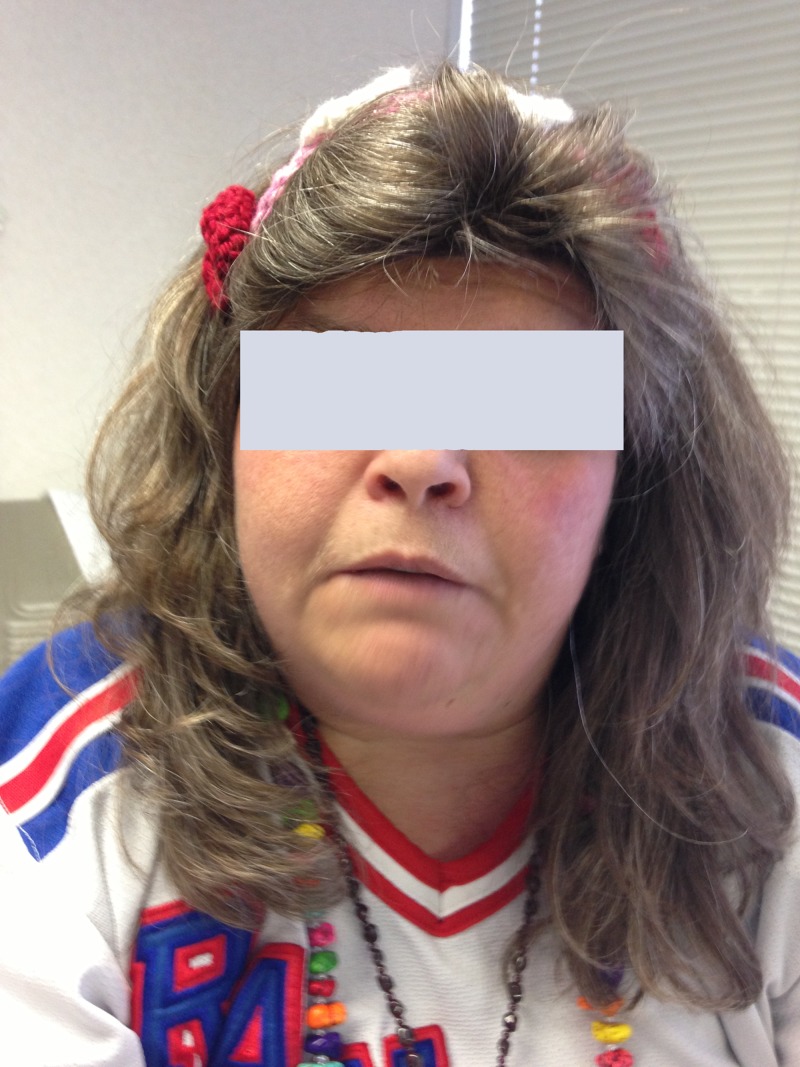
Picture of our patient showing round face.

**Figure 2:**
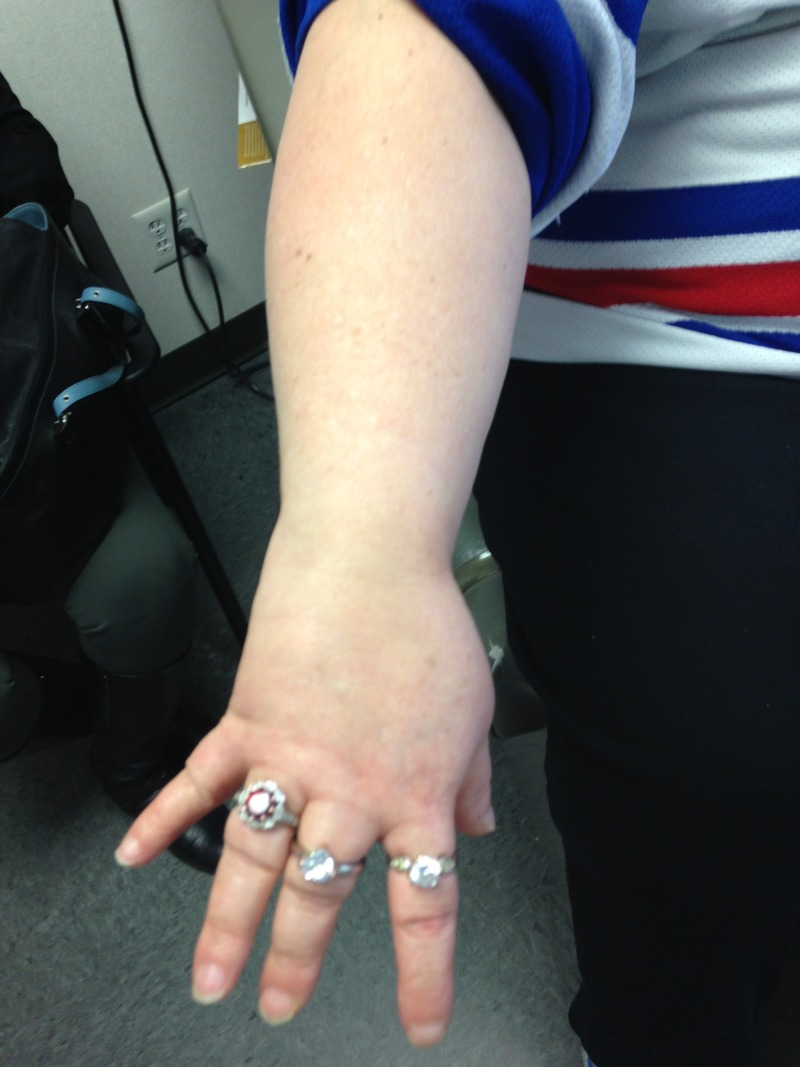
Brachydactyly in 3rd and 4th fingers.

Figure 1 shows round face of our patient and Figure 2 shows the brachydactyly of third and fourth fingers.

## Discussion

BDMR, also known as AHO-like syndrome and 2q37 deletion syndrome, is a rare disease that clinically resembles AHO. BDMR is a result of a chromosomal deletion on the long arm at chromosome 2q37 that gives it its name: 2q37 deletion syndrome [2]. Depending on each individual, sizes of these deletions may vary around 3.5–8.8 Mb, which can affect the presentations of this disease, and the severity of the condition in the patient [3]. BDMR is diagnosed after a child is born, usually during early developmental stages, and does not show signs in vitro. It can be characterized by specific craniofacial and skeletal abnormalities, such as round face, prominent forehead, flat nasal bridge, thin upper lip, Brachydactyly Type E and short stature [4]. It was suggested by Syrrou et al. that Glypican1 gene, responsible for neural and skeletal development, was the main candidate gene for Brachydactyly Type E which is a phenotype characterized by shortening of metacarpals [5]. Central nervous system, ocular, cardiac, gastrointestinal, renal, and other genitourinary anomalies have been noted in about one‐third of the patients. Patients with proximal breakpoint at band 2q37.1 manifest with Wilms tumor, renal dysplasia, and tracheomalacia and those with breakpoints throughout the region present with gastrointestinal anomalies, pyloric stenosis, and diaphragmatic defects [6]. These characteristics, along with delays in social skills, are: hyperactivity, rocking movements, psycho-social retardation, and even neurological disorders such as epilepsy [4]. Facial features are clearly present in this patient and many others, with symptoms similar to that of the patient’s, including round face, thin upper lip, slanted eyes, and depressed nasal bridge. This is an important marker for this disease because it differentiates from PPHP in physical traits, and craniofacial abnormalities can be easily spotted by trained healthcare professionals, which can lead to correct diagnosis [7]. Brachymetaphalangism, which refers to shortening of either the metacarpals and phalanges of the hands or bones in the feet, has been reported in approximately 50% of cases and congenital heart anomalies are present in around 20% of patients.

The main candidate gene for the brachymetaphalangism seen in BDMR is histone deacetylase 4 (HDAC4) on chromosome 2q37.2. HDAC4 acts as a transcription repressor by altering chromatin structure and is essential for brain, muscle and bone development, as well as function [8]. Studies also show that HDAC4 regulates chondrogenesis and skeletogenesis by inhibiting the activity of RunX-2 which is necessary for chondrocyte hypertrophy [9]. Wilson et al. suggested that there are three groups of patients in whom cytogenetics and molecular investigation of 2q37 will be valuable. They are those with brachymetaphalangy and mental retardation, those with AHO phenotype and normal GS alpha protein levels and those with brachydactyly type E and acrodysostosis [10].

Gsα expression is biallelic in most tissues and inactivating Guanine nucleotide protein-alpha subunit (GNAS) mutations on either the paternal or maternal allele results in Gsα deficiency leading to AHO and the resistance of target organs to PTH and other hormones which act through cAMP (cyclic adenosine monophosphate) only if the mutations are on the maternal allele [11,12].

Because the phenotype of BDMR is so variable, patients can be misdiagnosed if physicians are not looking at the chromosomes to research the deletion possibility. Additionally, social skills can greatly improve with age, making this disease even more difficult to diagnose in later years. However, early intervention has been proven to help patients see positive results earlier. This disease can be managed with the help from healthcare professionals, especially with periodic checkups to monitor any changes, and to combat the symptom of obesity in BDMR patients. Neurodevelopmental and behavioral evaluations, skeletal survey, endocrine evaluation, and an echocardiogram are indicated.

On review of the literature, we found the following studies and case reports regarding BDMR as shown below in Table 1.

**Table 1:** Review of literature of brachydactyly mental retardation syndrome. FISH: Fluorescence in situ hybridization; FARP2: Pleckstrin domain protein 2; HDLBP: Vigilin; PASK: Proline-alanine-rich STE20-related kinase; BDMR: Brachydactyly mental retardation syndrome; GNAS1: Guanine nucleotide protein-alpha subunit 1; HDAC4: Histone deacetylase 4; AHO: Albright hereditary osteodystrophy.

AUTHOR	STUDY INTRODUCTION	STUDY SUMMARY
Felder et al. [2]	This is a case report on a patient with 2q37.3 terminal deletion who had autism and brachymetaphalangy. FISH analysis and microsatellite genotyping were performed on this patient to precisely locate the deleted region.	An expression analysis of five candidate genes was performed to test the hypothesis that haploinsufficiency of genes located in the deleted region was responsible for this patient’s phenotype. The study found out that the expression of three genes FARP2, HDLBP, and PASK was downregulated but the extent to which this contributed to the patient’s symptoms was not clear.
Wheeler et al. [3]	This is a case report on three individuals with haploinsufficiency of HDAC4 who presented with Brachydactyly E, non-dysmorphic facial features and normal intelligence which is in contrast to mental retardation seen in BDMR patients with haploinsufficiency of HDAC4.	The study after reviewing various literatures pertaining to haploinsufficiency of HDAC4 came to the conclusion that isolated haploinsufficiency of HDAC4 cannot be the sole reason for intellectual disability in BDMR patients.
Shrimpton et al. [13]	This is a study involving three patients with Albright hereditary osteodystrophy-like phenotype, with deletions on 2q37.3. The deleted region included G-protein-coupled receptor 35 (GPR35), glypican 1 (GPC1), and serine/threonine protein kinase 25(STK25) genes.	The study suggested that patients with AHO should have a karyotype and sub-telomeric fluorescence in situ hybridization (FISH) studies performed and if patients lack GNAS1 mutations or 2q37.3 deletions, haploinsufficiency for GPR35 should be considered as the cause for 2q37-linked AHO and BDMR.
Morris et al. [14]	This is a case report of familial BDMR including a parent with mild BDMR and a child with a more severe phenotype.	Gene expression studies showed reduced expression of HDAC4 in both parent and offspring but its expression was reduced to <50% in the offspring. This led to the hypothesis that severity of BDMR may be influenced by the percentage of HDAC4 expression, where greater reduction in HDAC4 leads to more severe phenotype.
Tammachote et al. [15]	This is a case report on a girl who had phenotypes of both Primary hyperoxaluria 1 and BDMR.	The case report showed that the patient had a paternal de novo terminal deletion of chromosome 2q involving HDAC4 in one allele and Alanine:Glyoxylate Aminotransferase gene in another allele which was responsible for the primary hyperoxaluria phenotype.
Imitola et al. [16]	This is a case report about a patient with neurodevelopmental delay, microcephaly and seizures with a small 2q37 interstitial deletion.	The deleted segment in 2q37 included neural progenitor genes essential for the development of human cortex and corpus callosum. The gene STK25 present in the deleted segment is highly interacting and was shown to play a major role in brain development. Considering this, the study suggested that the deletion of STK25 and other nearby neuroprogenitor cells could be responsible for neurodevelopmental delay and microcephaly.
Phelan et al. [17]	This is a case report involving four unrelated individuals with AHO-like phenotype and 2q37.3 deletion.	The study suggested that there could be a possibility of a second disease locus, given the heterogeneity of the disease presentation.

## Conclusions

BDMR clinically resembles AHO and can be easily misdiagnosed as PPHP. Most cases are recognized during childhood, however, the diagnosis of BDMR should be considered in adults with AHO-like phenotype and normal calcium metabolism. It should also be considered in patients with mental retardation and normal calcium metabolism. While there are few cases of BDMR reported worldwide, we must anticipate there are more than this because of the hereditary nature of the disease and the frequency that these cases have been reported in a short period of time. Though BDMR is becoming more well-known in the medical community, there are no known preventions or cure for it yet. However, genetic counseling, diagnosis, and surveillance of BDMR are essential for a patient to live a healthy life.
